# Cases Treated at the Woman’s Hospital of the State of Illinois

**Published:** 1872-08-15

**Authors:** J. G. Brown

**Affiliations:** Resident Physician


### Original Communications.

### CASES TREATED AT THE WOMAN'S HOSPITAL OF THE STATE OF ILLINOIS.

REPORTED BY MRS. J. G. BROWN, RESIDENT PHYSICIAN.

CASE I. *Chlorosis complicated with dysmenorrhœa and leucorrhœa.*

K. L., aged twenty years; unmarried; was admitted February 10, 1872. She is a slight, pale woman, but never had any serious illness until about three months ago. Menstruation commenced at the age of fourteen, and continued normal in all respects to December last. Since that time the flow has rapidly diminished, and now is not more than one-fourth its usual amount. The discharge is accompanied by extreme pain, which is referred to the lumbar and pelvic regions. In the intervals she has a copious leucorrhœal discharge. Her general aspect is feeble, and her complexion waxy and pallid, with a slight tinge of green. Her appetite is irregular ; bowels constipated. She has occasional attacks of palpitation of the heart on making any exertion. There is also a condition of extreme nervous disquietude. Auscultation fails to reveal any organic disease of the heart.

The patient is one of the many whose circumstances have been changed by the great fire of last October. Since that event her hygienic surroundings have been of the worst character. Her home has been with a kind-hearted, indigent woman, into whose house three things essential to health, namely, nourishing diet, pure air and sunshine, never come. These were provided for her, and she was ordered to take ten-drop doses of the tincture of the chloride of iron four times daily. She was likewise ordered to have very warm sitz baths every second day, and to use an astringent vaginal lotion daily. Her bowels were to be kept regular by means of mild aperients and enemata.

March 6th. In every respect she is improved. Her bowels are regular, and appetite good. Her color has greatly improved. Her tongue is slightly furred, and she has diurnal headache. Sulphate of quinia is added to the iron, and she is to continue the rest of the treatment.

May 8th. Has so far regained her health as to be able to resume work, and is discharged.

*Remarks:* This case shows how rapidly bad hygienic influences, combined with the depressing effects of misfortune, may affect the health unfavorably. The patient, although not robust, had yet always been in fair health, but three months of life in a tainted atmosphere, with no conveniences for bodily cleanliness, had been sufficient to bring on serious general and local disorder. It also shows the rapidly beneficial effects of restorative medicine in such cases, when combined with proper attention to hygienic measures.

CASE II. *Obstinate Pruritus of the Vulva successfully treated by the Sulpho-Carbolate of Zinc.*

Mrs. H. L., aged 39 years, was admitted June 5, 1872. She commenced menstruating at the age of fifteen, the discharge being irregular, profuse, and attended with extreme pain, down to the time of her marriage, at the age of twenty-three. She is the mother of six children, the youngest being eighteen months old.

An examination revealed the existence of chronic endometritis, with extensive granular ulceration of the cervix uteri, and an abundant leucorrhœa. There was also an abscess in the right axilla, and another in the left groin. The bowels were constipated; the urine highly colored and scanty, but voided without pain or difficulty.

Notwithstanding her body seemed such a "Pandora's Box," she was insensible to all except the pruritis. This was described by her as far more terrible than any pain, being aggravated towards evening, and filling her nights with misery, and her days with dread of their return. It was for this only that she sought relief.

The cause of the pruritis was not quite clear. A slight papular eruption was visible about the parts, but this seemed dependent upon the use of some irritating applications that had been employed to allay the itching, since it disappeared on their withdrawal. It was probably produced by the acrid discharges from the inflamed uterus. This appeared more likely from the fact that when the leucorrhœal discharge was kept from the parts by protecting the latter with unctuous substances, syringing the vagina frequently, and the use of a tampon of cotton, saturated with glycerine, her sufferings were mitigated, although not wholly relieved.

Appropriate treatment was addressed to the general condition of the patient, and Dr. Jackson advised the use of a solution of the sulpho-carbolate of zinc as a local application for the pruritis. The vulva, after being thoroughly bathed with tepid water, was washed twice daily with a solution containing half a drachm to the ounce of distilled water, the parts being allowed to dry without wiping. She was much improved at the end of a week. During the second week the application was made once daily, on retiring at night. At the end of the third week the patient was summoned to Wisconsin by a death in her family, and at the time of leaving the hospital the pruritis was wholly relieved. Recent intelligence from her assures us that there has been no return, except a slight irritation during menstruation.

The sulpho-carbolate of zinc has been used in several other similar cases, and always with good results.

CASE III. *Amenorrhœa from undersized uterus.*

M. G., aged 32, was admitted June 5th, 1872. She has been married six years and has never conceived. Her habit is spare, and she has a weary, worn look, and a sallow complexion. The catamenia appeared at fourteen, and was natural until the time of her marriage. Since that time the flow has steadily diminished, until latterly it has amounted to only a few drops.

On examination, the uterus was found considerably under size, the cervix small and pointed, and the os so small as barely to admit the smallest uterine probe. From this latter there exuded a glairy, tenacious discharge. There was considerable leucorrhœa, both vaginal and uterine.

The treatment consisted in means calculated to induce uterine congestion and development. The cervix was painted freely with Churchill's tincture of iodine, and its canal slowly dilated by means of tents and the uterine dilator. Tepid sitz baths, and stimulating vaginal injections were used daily. She also took small doses of the syrup of the iodide of iron, three times daily, during the fortnight preceding the menstrual period.

Mrs. G. has now passed the third period since the above treatment was instituted. The uterus has become perceptibly larger, the menstrual flow has returned to its normal quantity, and her general health is greatly improved.

